# Integrative physiological study of adaptations induced by aerobic physical training in hypertensive hearts

**DOI:** 10.3389/fphys.2022.920196

**Published:** 2022-08-19

**Authors:** Suenimeire Vieira, Bruno A. Aguilar, Ana Catarine Veiga, Stella V. Philbois, Ana Caroline S. Freitas, Karine P. Rodrigues, Jens Tank, Hugo C. D. Souza

**Affiliations:** ^1^ Department of Physiology, Ribeirão Preto Medical School, University of São Paulo, São Paulo, Brazil; ^2^ Institute of Aerospace Medicine, German Aerospace Center, Cologne, Germany

**Keywords:** hypertension, autonomic nervous system, cardiac adaptations, physical training, experimental study

## Abstract

Aerobic physical training reduces arterial pressure in patients with hypertension owing to integrative systemic adaptations. One of the key factors is the decrease in cardiac sympathetic influence. Thus, we hypothesized that among other causes, cardiac sympathetic influence reduction might be associated with intrinsic cardiac adaptations that provide greater efficiency. Therefore, 14 spontaneously hypertensive rats (SHR group) and 14 normotensive Wistar Kyoto rats (WKY group) were used in this study. Half of the rats in each group were trained to swim for 12 weeks. All animals underwent the following experimental protocols: double blockade of cardiac autonomic receptors with atropine and propranolol; echocardiography; and analysis of coronary bed reactivity and left ventricle contractility using the Langendorff technique. The untrained SHR group had a higher sympathetic tone, cardiac hypertrophy, and reduced ejection fraction compared with the untrained WKY group. In addition, reduced coronary bed reactivity due to increased flow, and less ventricular contractile response to dobutamine and salbutamol administration were observed. The trained SHR group showed fewer differences in echocardiographic parameters as the untrained SHR group. However, the trained SHR group showed a reduction in the cardiac sympathetic influence, greater coronary bed reactivity, and increased left intraventricular pressure. In conclusion, aerobic physical training seems to reduce cardiac sympathetic influence and increase contractile strength in SHR rats, besides the minimal effects on cardiac morphology. This reduction suggests intrinsic cardiac adaptations resulting in beneficial adjustments of coronary bed reactivity associated with greater left ventricular contraction.

## 1 Introduction

Systemic arterial hypertension is accompanied by a progressive increase in cardiac sympathetic autonomic drive, which contributes to the early development of heart failure (Borovac et al., 2020; Seravalle and Grassi, 2022). This sympathetic hyperactivity aims to maintain adequate cardiac output regardless of the individual’s metabolic demand; although, other causes may be involved (Saxena et al., 2018). However, if the increase in arterial pressure persists, cardiac adaptations will become progressively relevant because of the significant augmentation of the sympathetic drive to the heart, resulting in adverse cardiac remodeling associated with reduced functional capacity (Floras, 2009; Oparil et al., 2018). Usually, this progression is silent, and in many patients, it is only noticed when damage to the heart is already at an advanced stage.

Conservative treatment is provided by administering different classes of drugs to reduce the preload and afterload (Diaconu et al., 2018). Nonetheless, new guidelines for managing hypertension recommend initiating treatment through regular aerobic physical exercises, especially in patients with grade I and II hypertension (Williams et al., 2018). However, the effects and mechanisms through which physical exercise influences hypertensive hearts remain unclear. More precisely, little is known about the relationship between cardiac sympathetic autonomic influence, coronary bed reactivity, and left ventricular contractility in hypertensive hearts, as well as the effects of aerobic physical training on these parameters. The literature suggests that long-term aerobic physical training can improve coronary flow regulation. However, little is known about this and the relationship between this improvement and cardiac functionality, especially left ventricular contractility (Laughlin et al., 1998; Jakovljevic et al., 2019). Therefore, we aimed to investigate the different aspects of cardiac adaptations promoted by aerobic physical training in spontaneously hypertensive rats (SHR) in an integrated way, focusing on autonomic control, coronary bed reactivity, and effects on left ventricular contractility, cardiac morphology, and function.

## 2 Materials and methods

### 2.1 Animals

Research trials were conducted on 18-week-old male Wistar Kyoto rats (WKY) and SHR. They were assigned to the following four groups: WKY untrained (*N* = 07), WKY trained (*N* = 07), SHR untrained (*N* = 07), and SHR-trained (*N* = 07). The rats were supplied by the Animal Facility of the Ribeirao Preto Medical School, University of São Paulo, Ribeirão Preto. They were housed in a room with strictly controlled temperature (21 ± 1°C) and a 12-h light/dark cycle, with unrestricted access to tap water and standard rat food (Nuvilab CR-1; Nuvital, Curitiba, Brazil). All experimental protocols performed in the current study were approved by the Committee on Animal Research and Ethics of Ribeirão Preto Medical School, University of São Paulo (Protocol 044/2020). This study was conducted in compliance with the ARRIVE guidelines.

### 2.2 Physical training

Aerobic physical training was carried out as previously described (Gardim et al., 2021). The rats in the training groups underwent aerobic physical training consisting of swimming sessions. The training program was conducted in two different stages over a total of 14 weeks. The first stage consisted of a 2-weeks adaptation period, during which the session length was gradually increased from 5 to 40 min/day, 5 times/week (in increments of 5 min/day). The second stage consisted of 12 weeks, with 40 min of physical training sessions conducted 5 times/week.

### 2.3 Experimental protocol

#### 2.3.1 Echocardiography

Echocardiography was performed as previously described (Felix et al., 2018). At 32 weeks of age, the rats were evaluated by echocardiography using a Vevo 2,100^®^ High-Resolution Imaging System (Visual Sonics, Toronto, ON, Canada) with a high-resolution transducer at 21 MHz. All animals were anesthetized with 1.5% isoflurane supplemented with 1% O_2_ and placed on a heated platform (37°C). Electrocardiogram and platform temperature were monitored. All measurements were performed according to the American Society of Echocardiography standards by a single individual, blinded to the characteristics of each group (Sahn et al., 1978).

#### 2.3.2 Recording of arterial pressure and heart rate

Forty-eight hours after the echocardiographic examination, the animals were anesthetized with ketamine and xylazine (80 mg/kg and 10 mg/kg, respectively). A polyethylene catheter (PE-50 soldered to PE-10; Intramedic, Clay Adams, Parsippany, NJ, United States) was implanted into the left femoral artery to record the hemodynamic parameters, systolic arterial pressure (SAP), diastolic arterial pressure (DAP), and mean arterial pressure (MAP). The catheters were tunneled subcutaneously, exteriorized at the nape, and filled with a heparinized saline solution (500 IU/ml) to prevent blood clotting.

Arterial pressure (AP) pressure was recorded in conscious rats kept in a quiet environment, using a pressure transducer (MLT0380, ADInstruments, Bella Vista, Australia), and the amplified signal (ML110, ADInstruments) was fed to a computer acquisition system (LabChart 7 Pro, ADInstruments). MAP and heart rate (HR) were calculated from arterial pulse pressure.

#### 2.3.4 Cardiac sympathovagal balance after double blockade of autonomic receptors

Cardiac sympathovagal balance was assessed as previously described (Gardim et al., 2021). Twenty-4 hours after the surgical procedure, the femoral artery catheter was attached to a pressure transducer and pulsatile AP was sampled continuously (2 kHz). MAP and HR were calculated from pulsatile AP using a software program (PowerLab, LabChart8; ADInstruments).

Cardiac autonomic receptor blockade (both sympathetic and vagal) of HR was assessed by administering atropine (4 mg/kg) and propranolol (5 mg/kg). After the basal HR was recorded for 30 min, atropine was injected into half of the animals in each group. The HR was recorded for the next 15 min to assess the effect of muscarinic receptor blockade on the HR (sympathetic effect). Propranolol was subsequently injected into the same animals, and the HR was recorded for the next 15 min to determine intrinsic HR (iHR). In half of the animals, the atropine-propranolol sequence was reversed to a propranolol-atropine sequence, following the same recording procedure (15 min) for each drug used in the previous sequence to determine iHR. After 24 h the same procedure was performed, however the sequence of drugs was inverted in each animal.

#### 2.3.5 Preparation of isolated hearts for perfusion—langendorff technique

Isolation of hearts for perfusion was performed as previously described (Felix et al., 2018). The hearts were perfused (8 ml/g) with Krebs–Henseleit solution and gassed with a combination of 95% O_2_ and 5% CO_2_ at 37°C. Coronary perfusion and left intraventricular pressure were measured using a latex balloon inserted into the left ventricle (LV). After 10 min of baseline stabilization, coronary bed reactivity was evaluated by gradually increasing the basal flow rate by 20% every 2 min until it reached 200%. The following parameters were recorded for each flow rate: coronary perfusion pressure, maximum left intraventricular pressure, maximum systolic contraction velocity (dP/dT_max_), and maximum diastolic relaxation velocity (dP/dT_min_). β-Adrenergic receptor sensitivity was evaluated using the cardiac dose-response curve, and the maximum intraventricular pressure response was obtained by the *bolus* administration of dobutamine (β1-adrenergic agonist, 0.5–50 nmol) and salbutamol (β2-adrenergic agonist, 5–100 nmol).

### 2.4 Statistical analysis

The results are presented as mean ± standard error. The effects of hypertension and physical training were assessed using a two-way analysis of variance. When appropriate, post-hoc comparisons were performed using the Student–Newman–Keuls test. The reactivity of the coronary vascular bed and dose-response curves of cardiac contractility after dobutamine and salbutamol administration were analyzed using a multivariate repeated-measures model, and post-hoc comparisons were performed when appropriate. Differences were considered statistically significant at *p* < 0.05. All statistical tests were performed using the SigmaPlot 11.0 software (Systat Software Inc., San Jose, CA, United States).

## 3 Results

Table 1 shows the values of hemodynamic parameters for all groups and the HR values before and after the double blockade of cardiac autonomic receptors with atropine and propranolol. The SHR group had higher BP than the normotensive group. The trained SHR group had lower AP than the untrained SHR group, while both WKY groups (untrained and trained) did not differ in their AP.

**TABLE 1:** Hemodynamic parameters and values of heart rate (HR) obtained before and after the double pharmacological blockade of cardiac autonomic receptors with atropine and propranolol, in Wistar Kyoto (WKY, *N* = 14) and Spontaneously Hypertensive Rats (SHR, *N* = 14) groups, both untrained (*N* = 07) and trained (*N* = 07).

	WKY		SHR		Hypertension factor		Physical training factor		Interaction	
	Untrained	Trained	Untrained	Trained	F_(d.f)_	P	F_(d.f)_	P	F_(d.f)_	P
Hemodynamic Parameters										
SBP, mmHg	119 ± 3	118 ± 8	214 ± 3^*a,b*^	173 ± 6 ^*a,b,c*^	F_(1.27)_: 196.758	<0.001	F_(1.27)_: 15.909	<0.001	F_(1.27)_: 13.844	<0.001
DBP, mmHg	77 ± 8	76 ± 12	174 ± 4 ^*a,b*^	127 ± 20 ^*a,b,c*^	F_(1.27)_: 36.104	<0.001	F_(1.27)_: 3.681	0.067	F_(1.27)_: 3.422	0.077
MBP, mmHg	94 ± 6	93 ± 11	190 ± 3 ^*a,b*^	142 ± 18 ^*a,b,c*^	F_(1.27)_: 41.985	<0.001	F_(1.27)_: 4.776	0.039	F_(1.27)_: 4.446	0.046
Double autonomic blockade										
Basal HR, bpm	349 ± 6	326 ± 5 ^*a*^	391 ± 7 ^*a,b*^	335 ± 8 ^*a,b,c*^	F_(1.27)_: 15.568	<0.001	F_(1.27)_: 38.680	<0.001	F_(1.27)_: 6.572	0.017
Atropine, bpm	411 ± 6	395 ± 5	415 ± 7	390 ± 5 ^*c*^	F_(1.27)_: 0.007	0.934	F_(1.27)_: 11,750	0.002	F_(1.27)_: 0.438	0.515
Propranolol, bpm	326 ± 7	305 ± 6 ^*a*^	301 ± 5 ^*a*^	290 ± 7 ^*a*^	F_(1.27)_: 10.822	0.003	F_(1.27)_: 6.732	0.016	F_(1.27)_: 0.735	0.400
Intrinsic HR, bpm	359 ± 5	336 ± 4 ^*a*^	316 ± 4 ^*a,b*^	304 ± 3 ^*a,b,c*^	F_(1.27)_: 91.740	<0.001	F_(1.27)_: 23.100	<0.001	F_(1.27)_: 0.425	0.520

All values are presented as the mean ± SEM., SAP, systolic arterial pressure; mmHg, millimeter of mercury; DAP, diastolic arterial pressure; MAP, mean arterial pressure; HR, heart rate; bpm, beats per minute; F, factor; d.f, degrees of freedom. ^*a*^ *p* < 0.05 vs. WKY, untrained; ^*b*^ *p* < 0.05 vs. WKY, trained; ^*c*^ *p* < 0.05 vs. SHR, untrained.

The untrained SHR group had baseline tachycardia compared to the WKY group (391 ± 7 vs. 349 ± 6 bpm, *p* < 0.001). In contrast, both trained SHR (335 ± 8, *p* < 0.001) and WKY (326 ± 5 bpm, *p* < 0.021) groups showed bradycardia after aerobic exercise training. Administration of atropine promoted tachycardic responses in both untrained groups: WKY (411 ± 6 bpm) and SHR (415 ± 7 bpm). In a comparison of the untrained groups with their respective trained groups, the trained SHR group showed a lower tachycardic response (390 ± 5 vs. 415 ± 7 bpm, *p* < 0.005). Administration of propranolol promoted greater bradycardic responses in the untrained SHR group in compared to untrained WKY group (301 ± 5 vs. 326 ± 7 bpm, *p* < 0.003). Within the SHR groups, propranolol administration promoted similar bradycardic responses (301 ± 5 vs. 290 ± 7 bpm, *p* < 0.207, untrained and trained, respectively). Within the WKY groups, the WKY-trained group exhibited more prominent bradycardic responses (305 ± 6 vs. 326 ± 7 bpm, *p* < 0.021). Table 1 also shows the pacemaker iHR obtained after the double blockade of cardiac autonomic receptors. The untrained SHR group had a lower iHR than the untrained WKY group (316 ± 4 vs. 359 ± 5 bpm, *p* < 0.001). However, both trained groups, SHR (304 ± 3 bpm, *p* < 0.041) and WKY (336 ± 4 bpm, *p* < 0.001), showed a reduction in iHR.

**FIGURE 1:**
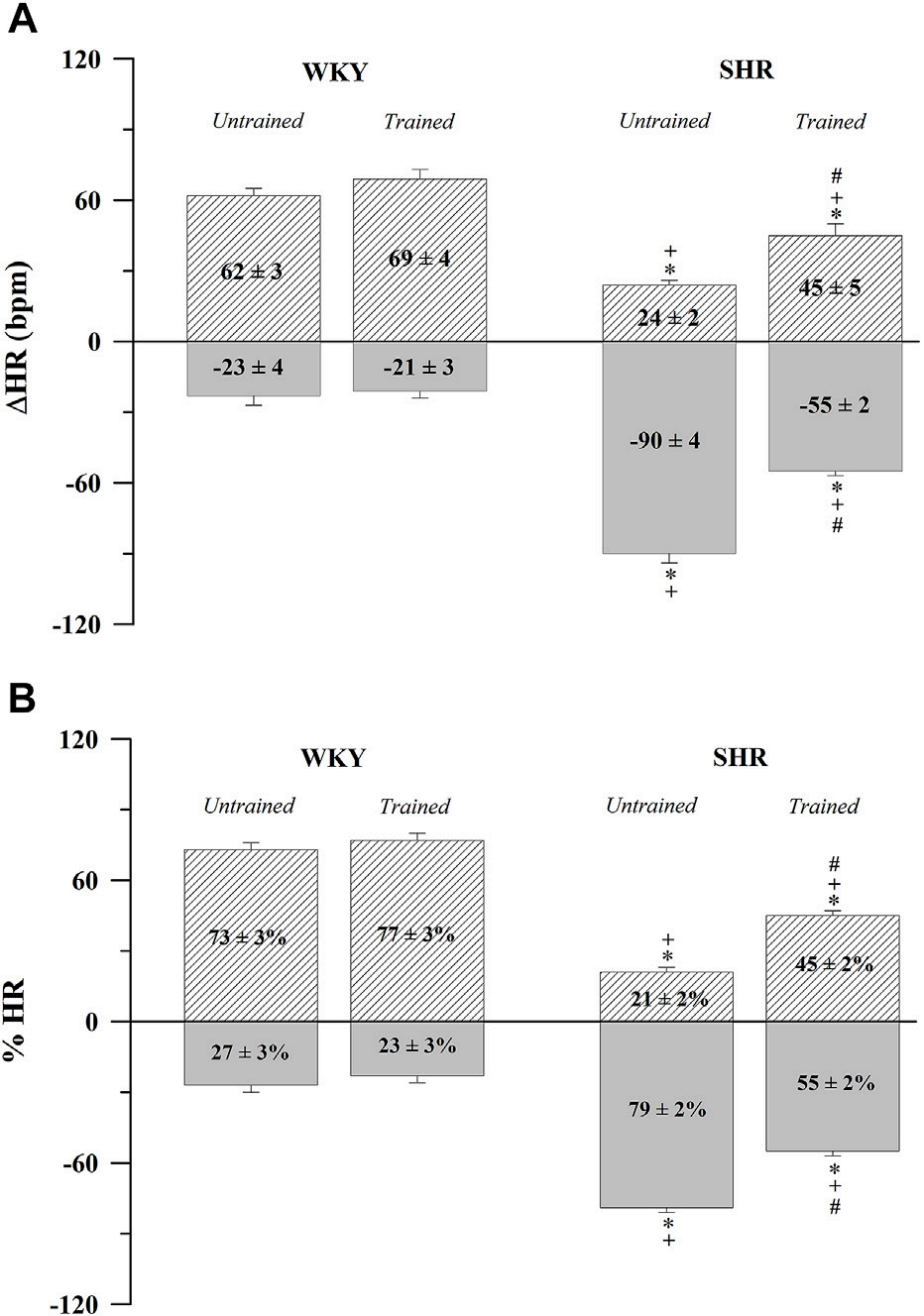
Absolute **(A)** and normalized **(B)** tonic autonomic cardiac responses to atropine (positive values) or propranolol (negative values) in trained (*N* = 07) and untrained (*N* = 07) normotensive Wistar Kyoto (WKY) and spontaneously hypertensive rats (SHR). Values are expressed as means ± standard error of the mean. *p < 0.05 vs. WKY untrained group; ^**+**^ *p* < 0.05 vs. WKY trained group; ^**#**^ *p* < 0.05 vs. SHR untrained group.

In contrast, Figure 1 shows the absolute values (Figure 1A) and percentages (Figure 1B) of the HR variations after the administration of atropine (positive values) and propranolol (negative values). The untrained SHR group had lower absolute (24 ± 2 bpm, *p* < 0.001) and percentage (21 ± 2%, *p* < 0.001) positive values and higher absolute (−90 ± 4 bpm, *p* < 0.001) and percentage (79 ± 2%, *p* < 0.001) negative values after the administration of atropine and propranolol, respectively, compared with the WKY and trained SHR groups. In summary, Figure 1 shows that the WKY group had a predominant vagal autonomic influence in determining the baseline HR, whereas the SHR group had a predominant sympathetic autonomic influence. The trained SHR group showed a reduction in sympathetic influence; however, sympathetic predominance persisted.

The body weight and cardiac morphological parameters of all groups are shown in Table 2. The untrained SHR group had lower body weight (302 ± 3 vs. 374 ± 9 g, *p* < 0.001) and higher absolute (1.8 ± 0.07 vs. 1.2 ± 0.07 g, *p* < 0.001) and relative heart weight (6.0 ± 0.06 vs. 3.2 ± 0.07 mg/g, *p* < 0.001), relative PWT (7.5 ± 0.35 vs. 4.1 ± 0.45 mm/kg, *p* < 0.001), IVST (6.6 ± 0.31 vs. 3.4 ± 0.47 mm/kg, *p* < 0.001), RWT (0.52 ± 0.02 vs. 0.43 ± 0.02 mm/kg, *p* < 0.008) LVEDD (28.4 ± 0.9 vs. 21.2 ± 2.2 mm/kg, *p* < 0.010), LVESD (18.6 ± 0.86 vs. 13.2 ± 1.7 mm/kg, *p* < 0.015), and LV mass (4.2 ± 0.2 vs. 2.3 ± 0.1 mg/g, *p* < 0.001) than the untrained WKY group. However, the trained WKY and SHR groups did not differ from their respective untrained groups.

**TABLE 2:** General characteristics and cardiac morphology parameters for the Wistar Kyoto (WKY, *N* = 14) and spontaneously hypertensive rats (SHR, *N* = 14) groups, both untrained (*N* = 07) and trained (*N* = 07).

	WKY		SHR		Hypertension factor		Physical training factor		Interaction	
	Untrained	Trained	Untrained	Trained	F_(d.f)_	P	F_(d.f)_	P	F_(d.f)_	P
Parameters										
Body weight, g	374 ± 9	357 ± 6	302 ± 3 ^*a,b*^	314 ± 7 ^*a,b*^	F_(1.27)_: 72.032	<0.001	F_(1.27)_: 0.115	0.737	F_(1.27)_: 4.592	0.042
Heart weight, g	1.2 ± 0.07	1.3 ± 0.14	1.8 ± 0.07 ^*a,b*^	1.8 ± 0.07 ^*a,b*^	F_(1.27)_: 36.181	<0.001	F_(1.27)_: 0.122	0.730	F_(1.27)_: 0.499	0.487
RHW, mg/g	3.2 ± 0.07	3.6 ± 0.08 ^*a*^	6.0 ± 0.06 ^*a,b*^	5.6 ± 0.07 ^*a,b,c*^	F_(1.27)_: 193.101	<0.001	F_(1.27)_: 0.042	0.984	F_(1.27)_: 32.397	<0.001
Cardiac Morphology										
PWT, mm/kg	4.1 ± 0.45	3.6 ± 0.38	7.5 ± 0.35 ^*a,b*^	7.0 ± 0.49 ^*a,b*^	F_(1.27)_: 366.79	<0.001	F_(1.27)_: 7.19	0.222	F_(1.27)_: 0.005	0.942
IVST, mm/kg	3.4 ± 0.47	3.5 ± 0.37	6.6 ± 0.31 ^*a,b*^	5.8 ± 1.1 ^*a,b*^	F_(1.27)_: 127.03	<0.001	F_(1.27)_: 2.069	0.163	F_(1.27)_: 2.593	0.120
RWT, mm/g	0.43 ± 0.02	0.42 ± 0.05	0.52 ± 0.02 ^*a,b*^	0.63 ± 0.05 ^*a,b*^	F_(1.27)_: 14.898	<0.001	F_(1.27)_: 1.594	0.219	F_(1.27)_: 2.295	0.143
LVEDD, mm/kg	21.2 ± 2.16	23.7 ± 2.7	28.4 ± 0.9 ^*a,b*^	28.1 ± 2.84 ^*a,b*^	F_(1.27)_: 45.41	<0.001	F_(1.27)_: 1.502	0.232	F_(1.27)_: 2.585	0.121
LVESD, mm/kg	13.2 ± 1.7	13.4 ± 1.5	18.6 ± 0.86 ^*a,b*^	17.5 ± 1.9 ^*a,b*^	F_(1.27)_: 69.494	<0.001	F_(1.27)_: 0.533	0.472	F_(1.27)_: 1.189	0.286
LV mass, mg/g	2.3 ± 0.1	2.2 ± 0.1	4.2 ± 0.2 ^*a,b*^	4.0 ± 0.1 ^*a,b*^	F_(1.27)_: 187.377	<0.001	F_(1.27)_: 0.807	0.378	F_(1.27)_: 0.137	0.715

All values are presented as the mean ± SD. g, grams; mg, milligrams; kg, kilograms; mm, millimeters; PWT, posterior wall thickness; IVST, interventricular septum thickness; RWT, relative wall thickness; LVEDD, left ventricular end-diastolic diameter; LVESD, left ventricular end-systolic diameter; LV, left ventricular; F, factor; d.f, degrees of freedom. ^*a*^ *p* < 0.05 vs. WKY, untrained; ^*b*^ *p* < 0.05 vs. WKY, trained.

Table 3 presents the results of the functional parameters. The results showed that the untrained SHR group had lower EF (75 ± 1 vs. 78 ± 2%, *p* < 0.032) and SF (35 ± 1 vs. 39 ± 2%, *p* < 0.014) values than the untrained WKY group. The trained WKY and SHR groups did not differ from their respective untrained groups.

**TABLE 3:** Cardiac function values obtained in the Wistar Kyoto (WKY, *N* = 14) and Spontaneously Hypertensive Rats (SHR, *N* = 14) groups, both untrained (*N* = 07) and trained (*N* = 07).

	WKY		SHR		Hypertension factor		Physical training factor		Interaction	
	Untrained	Trained	Untrained	Trained	F_(d.f)_	P	F_(d.f)_	P	F_(d.f)_	P
Cardiac Function										
HR, bpm	335 ± 8	315 ± 6 ^*a*^	360 ± 7 ^*a,b*^	339 ± 7 ^*b,c*^	F_(1.27)_: 17.287	<0.001	F_(1.27)_: 2.644	0.037	F_(1.27)_: 0.165	0.688
LVEDV, µl/g	1.40 ± 0.11	1.59 ± 0.12	1.39 ± 0.13	1.44 ± 0.26	F_(1.27)_: 0.181	0.674	F_(1.27)_: 0.535	0.471	F_(1.27)_: 0.219	0.644
LVESV, µl/g	0.30 ± 0.03	0.31 ± 0.04	0.35 ± 0.04	0.36 ± 0.05	F_(1.27)_: 1.532	0.228	F_(1.27)_: 0.389	0.845	F_(1.27)_: 0.290	0.958
Stroke volume, µl/g	1.08 ± 0.07	1.30 ± 0.15	1.04 ± 0.10	1.11 ± 0.16	F_(1.27)_: 0.876	0.359	F_(1.27)_: 1.356	0.256	F_(1.27)_: 0.339	0.566
Ejection fraction, %	78 ± 2	82 ± 2	75 ± 1 ^*a,b*^	77 ± 2	F_(1.27)_: 5.177	0.032	F_(1.27)_: 2.5	0.127	F_(1.27)_: 0.335	0.568
Shortening fraction, %	39 ± 2	43 ± 1	35 ± 1 ^*a,b*^	37 ± 2 ^*b*^	F_(1.27)_: 7.108	0.014	F_(1.27)_: 3.07	0.093	F_(1.27)_: 0.284	0.599
Cardiac output, mL/min	131 ± 9	143 ± 18	111 ± 6	119 ± 16	F_(1.27)_: 2.874	0.103	F_(1.27)_: 0.579	0.454	F_(1.27)_: 0.0302	0.863
Cardiac index, ml/g	0.35 ± 0.02	0.40 ± 0.02	0.37 ± 0.04	0.38 ± 0.02	F_(1.27)_: 0.306	0.956	F_(1.27)_: 1.225	0.279	F_(1.27)_: 0.689	0.415

All values are presented as the mean ± SEM., bpm, beats per minute; µL, microliters; %, percentage; mL, milliliters; min, minute; g, grams; HR, heart rate; LVEDV, left ventricular end-diastolic volume; LVESV, left ventricular end-systolic volume; F, factor; d.f, degrees of freedom. ^*a*^ *p* < 0.05 vs. WKY, untrained; ^*b*^ *p* < 0.05 vs. WKY, trained; ^*c*^ *p* < 0.05 vs. SHR, untrained.

**FIGURE 2:**
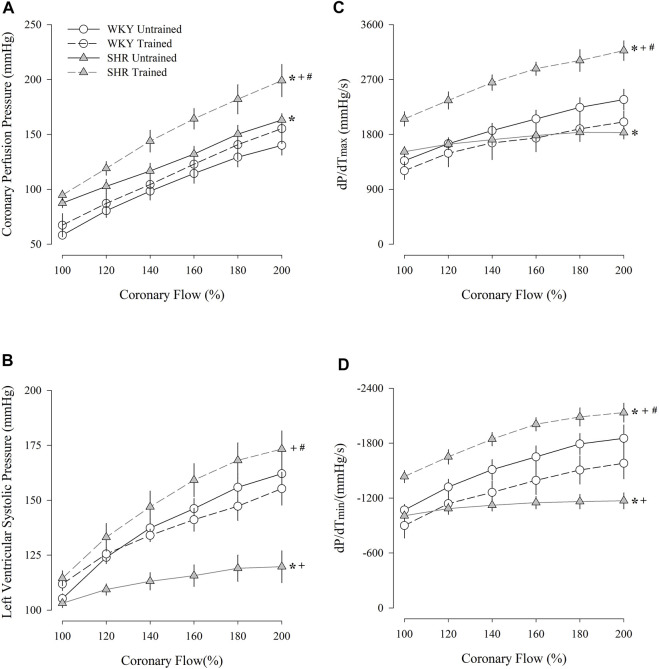
Coronary perfusion pressure **(A)**, left ventricular systolic pressure **(B)**, dP/dT_(max)_ **(C)**, and dP/dT_(min)_ **(D)** flow-induced in untrained (*N* = 07) and trained (*N* = 07) normotensive Wistar Kyoto (WKY) and hypertensive spontaneously rats (SHR). mmHg, millimeters of mercury; s, second; max, maximum; min; minimum. Values are expressed as means ± standard error of the mean. *p < 0.05 vs. WKY untrained group; ^**+**^ *p* < 0.05 vs. WKY trained group; ^**#**^ *p* < 0.05 vs. SHR untrained group.

Figure 2 shows the coronary perfusion pressure results (Figure 2A), left ventricular systolic pressure (Figure 2B), maximum dP/dT (Figure 2C), and minimum dP/dT (Figure 2D) in response to a progressive increase in flow. The untrained SHR group had higher values of coronary perfusion pressure and lower values of left ventricular systolic pressure and maximum and minimum dP/dT than the untrained WKY group. In contrast, the trained SHR group had higher values for all parameters than the untrained group. A comparison between both trained groups showed that the trained SHR group had the highest responses for all the parameters.

**FIGURE 3:**
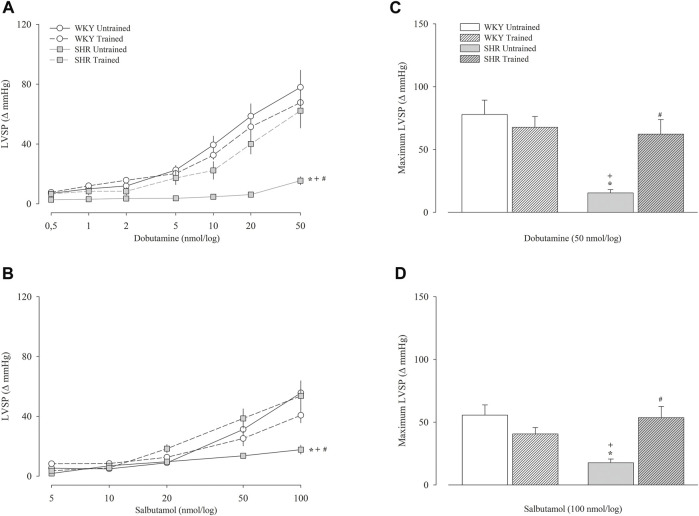
Left ventricular systolic pressure (LVSP) after administration of dobutamine **(A)** and salbutamol **(B)** in untrained (*N* = 07) and trained (*N* = 07) normotensive Wistar Kyoto (WKY) and hypertensive spontaneously rats (SHR). The graphs on the right represent the maximum response obtained for dobutamine (50 nmol) **(C)** and salbutamol (100 nmol) **(D)**. Values are expressed in delta (Δ). mmHg, millimeters of mercury. All values are presented as the mean ± standard error of the mean. *p < 0.05 vs. WKY untrained group; ^**+**^ *p* < 0.05 vs. WKY trained group; ^**#**^ *p* < 0.05 vs. SHR untrained group.

Figure 3 shows the variation in the Δ values of the left ventricular systolic pressure obtained after the administration of dobutamine (Figure 3A) and salbutamol (Figure 3B) and their respective maximal responses (Figures 3C,D, respectively). The untrained SHR group had lower left ventricular systolic pressure values for both dobutamine and salbutamol than untrained WKY group, including maximal response (dobutamine, 15 ± 3 vs. 78 ± 11 mmHg, *p* < 0.001); salbutamol, 18 ± 3 vs. 56 ± 8 mmHg, *p* < 0.001). Within the WKY groups, dobutamine or salbutamol promoted similar responses. In contrast, within the SHR groups, the trained SHR group had a significant increase, including in maximal responses to dobutamine (62 ± 12 vs. 15 ± 3 mmHg, *p* < 0.001) and salbutamol (54 ± 9 vs. 18 ± 3 mmHg, *p* < 0.001).

**FIGURE 4:**
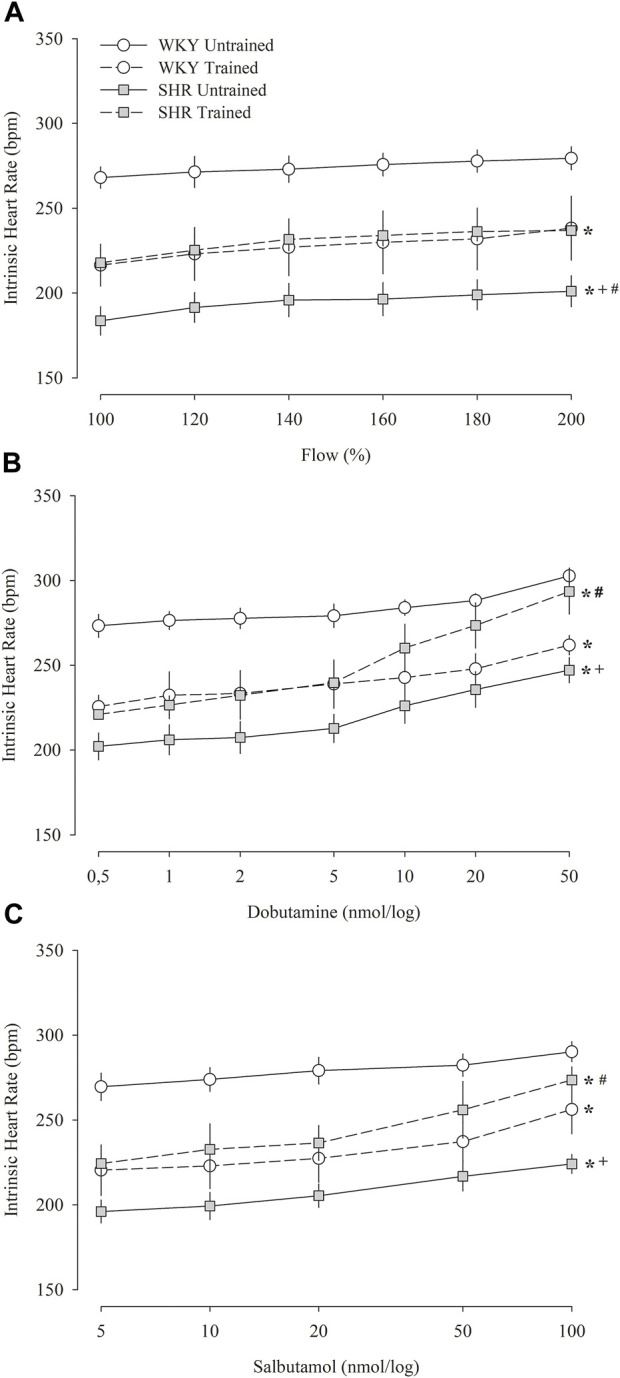
Intrinsic heart rate obtained in isolated hearts (Langendorff technique) during the percent increase in flow **(A)** and after multiple and sequential doses of dobutamine **(B)** and salbutamol **(C)** in untrained (*N* = 07) and trained (*N* = 07) normotensive Wistar Kyoto (WKY) and hypertensive spontaneously rats (SHR). bpm, beats per minute. All values are presented as the mean ± standard error of the mean. *p < 0.05 vs. WKY untrained group; ^**+**^ *p* < 0.05 vs. WKY trained group; ^**#**^ *p* < 0.05 vs. SHR untrained group.

Figure 4 shows the iHR responses of pacemakers in the isolated heart in relation to increased coronary flow (Figure 4A), administration of dobutamine (Figure 4B), and salbutamol (Figure 4C). Figure 4A shows that during the increase in flow percentage, the untrained WKY group had the highest iHR values, whereas the untrained SHR group had the lowest values. The trained groups showed similar values; however, compared to their respective untrained groups, the trained WKY group showed a reduction in iHR, while the trained SHR group showed an increase. Similar responses were observed in iHR after the administration of dobutamine (Figure 4B) and salbutamol (Figure 4C).

## 4 Discussion

The effects of aerobic physical training on cardiac autonomic control, morphology, and functionality were investigated in hypertensive rats. Our results suggested that untrained SHR had a greater sympathetic participation in determining the basal HR, which was associated with increased morphological parameters compared with untrained WKY rats. Our findings also showed lower coronary bed reactivity and reduced left ventricular contractility in response to increased flow or β-adrenergic agonist administration. Compared with untrained SHR, trained SHR showed a reduction in sympathetic participation on basal HR determination, and in the isolated heart had a greater coronary bed reactivity, and LV contractility in response to increased flow and dobutamine and salbutamol administration. However, we did not observe any significant modifications in the morphological parameters.

Double blockade of autonomic receptors with atropine and propranolol showed that untrained SHR had increased sympathetic participation in the determination of basal HR, despite reduced iHR. Therefore, together with cardiac hypertrophy, this may suggest important cardiac impairments (Tucker and Domino, 1988; Maskali et al., 2014). A reduction in iHR was also observed in the isolated hearts during the Langendorff protocol. However, while the iHR obtained in the double blockade of autonomic receptors was even more reduced in the trained SHR, the iHR obtained through the isolated heart was significantly increased compared to the untrained SHR; this is a paradox. The cause of this reduction in intrinsic pacemaker HR is unknown. A recent study on atrial preparations of SHR showed that the sympathetic and parasympathetic nerve terminals of the right atrium isolated from the SHR show a release of noradrenaline and acetylcholine in the absence of electric field and pharmacological stimulation. Nonetheless, this release of neurotransmitters does not regulate basal chronotropism. In this case, the reduced chronotropism in atrial preparations and in isolated heart from SHR should not involve neurogenic changes and is probably mediated by intrinsic changes in the atrial tissue. (Rodrigues et al., 2019). In contrast, our findings suggest that aerobic physical training contributes to adjustments in pacemaker intrinsic heart rate regulation.

Regarding cardiac hypertrophy, the effects of hypertension and chronic elevation of sympathetic activity on the heart are supported by our results. The SHR group had lower body mass and significantly higher heart mass than the WKY group, even in absolute values. Furthermore, despite normalizing by body mass, the heart mass values of the SHR group were twice those of the WKY group. This result was consistent with the morphological values obtained through echocardiography, which were significantly higher in the hypertensive group than the normotensive group for all parameters. On the other hand, although the heart of hypertensive animals presented significantly higher dimensions than normotensive animals, such differences were not observed in functionality, except for a slight reduction in the ejection fraction. Therefore, the SHR group presented values of cardiac functionality similar to those of normotensive animals.

The cardiac morphological changes observed in hypertensive animals occur in response to the high peripheral resistance of the cardiac muscle, which reacts to such an overload by addition of sarcomeres in parallel (Yildiz et al., 2020). In the first weeks of life, the animal’s heart manages through a hypertrophic response and other adaptations to preserve the parameters of cardiac functionality. However, after a longer period of pressure overload, the cardiac functionality parameters of hypertensive animals are impaired, which is due to a progressive inflammatory state that is sustained by adaptations promoted by the pathology itself (Conrad et al., 1991; Conrad et al., 1995; Xu et al., 2020). However, in our case, the animals were only 32 weeks old.

The study of isolated hearts allowed us to evaluate the cardiac parameters without directly influencing the autonomic nervous system. The high values of coronary perfusion pressure observed in the trained SHR group coincided with an increase in LV contraction strength and suggested a greater self-regulatory capacity of the coronary arteries. Increased LV contraction strength promotes greater pressure in the cardiac vascular beds, which hampers the flow from passing (Duncker and Bache, 2008). In contrast, normotensive animals did not show significant differences in LV systolic pressure, which supports the lack of any observed change in coronary perfusion pressure in the trained group.

Furthermore, coronary perfusion pressure was higher in hypertension-trained animals than in sedentary animals. The increase in perfusion pressure is due to an increase in flow, which is associated with an autoregulatory response to the constriction of the arterioles (Cowley, 1980; Goodwill et al., 2017). In this sense, the results indicate that aerobic physical training promotes an improvement in the self-regulatory mechanism of the coronary bed in hypertensive animals. The fact that physical training led to a similar improvement in both untrained and trained WKY rats can be attributed to the already adequate functioning of the coronary bed in these animals. Additionally, other studies that compared cardiovascular adaptations observed in SHR and WKY rats under different physical training protocols demonstrated that SHR rats are more susceptible to physical training owing to their impaired vascular condition. (Pagan et al., 2015).

Left ventricular systolic pressure refers to the maximum force of contraction produced by the myocardium, whereas dP/dT_max_ represents the maximum increase in intraventricular pressure and is associated with cardiac contractility (Frank and Levinson, 1968; Hamlin and del Rio, 2012). The reduction of such parameters, mainly at higher flow percentages, indicates losses in the physiological reserve for increases in strength and contraction speed and is often attributed to the proteins responsible for regulating calcium in cardiomyocytes (Carneiro-Júnior et al., 2013; Carneiro-Júnior et al., 2014). However, when subjected to the aerobic physical training protocol, the values of systolic pressure of the LV, dP/dT_max_, and HR approached the values obtained in the normotensive groups, indicating important adaptations in the cardiac tissue. These results are supported by findings from other studies which indicate that among other adaptations, aerobic physical training in SHR promotes an improvement in the regulation of proteins involved in the processes of formation/degradation of collagen fibers, contractile function, and autonomic dysfunction (Masson et al., 2015; Locatelli et al., 2017; Pagan et al., 2019). The left ventricular relaxation rate was assessed using dP/dT_min_. The results showed that hypertensive animals had a reduced dP/dT_min_ compared to normotensive animals, suggesting that the relaxation velocity of the left ventricular muscles was lower. These animals also presented with a high baseline HR indicating that the time available for myocardial relaxation is too short, which may in turn contribute to a lower end-diastolic volume. In addition, when we analyzed the delta variation of dP/dT_min_ in response to flow, we noticed no changes in response to the increase in flow, indicating that the maximum velocity of ventricular relaxation is within the physiological limits of the basal flow. These results are consistent with the published findings on left ventricular diastolic dysfunction in the respective lineage of animals, which is supported by the observed changes in intracellular calcium reuptake after cardiomyocyte excitation (Dupont et al., 2012). The physical training protocol in hypertensive animals promoted an increase in dP/dT_min_ and the response to flow variation, which may be associated with an improvement in intracellular calcium reuptake as demonstrated in other studies (Rolim et al., 2007; Yoshizaki et al., 2018).

In conclusion, our results showed that the reduction in the cardiac sympathetic autonomic effect induced by aerobic physical training was accompanied by an increase in left intraventricular pressure, suggesting recovery of intrinsic cardiac properties, such as contractility when analyzed by Langendorff technique. In contrast, the echocardiography did not show differences.

### 4.1 Study limitations

The echocardiographic recordings were performed with anesthetized animals, and these may have interfered with our results. We did not measure plasma catecholamines or cardiac norepinephrine spillover as more direct parameters to characterize central sympathetic drive and cardiac tone. Swimming training model is a less frequent training than motorized treadmill. However, satisfactory levels of aerobic physical training have been achieved according to the observed results. Another limitation was the choice to investigate only male rats. Although systemic arterial hypertension indiscriminately affects both the sexes, we are convinced that female SHR need to be exclusively studied to address hormonal issues, since ovarian hormones seem to participate directly and indirectly in cardiovascular autonomic regulation and influence cardiac morphology and functionality.

## Data Availability

The raw data supporting the conclusion of this article will be made available by the authors, without undue reservation.
